# Hypnotism

**Published:** 1860-03

**Authors:** 


HYPNOTISM.—Considerable excitement has been produced in Paris during the past few months, by the application of hypnotism to surgical operations, and the subject was presented to the Academy of Sciences by M. Velpeau as a new discovery; but upon investigation, it was found that it was merely the revival of a method suggested ten years ago by Dr. Braid, of England. It consists in holding a bright object a few inches in front of the eyes, so that they are directed upon it while the superior recti muscles are contracted to their utmost extent. Convergent strabismus is produced; the patient falls into a cataleptic condition, and anæsthesia results, which lasts a few minutes, but which is interrupted by slight noise or by the movement of the object before the eyes.

MM. Broca and Follin had under their care a patient affected with perineal abscess, which was opened under the influence of hypnotism, without any pain. From a large number of experiments conducted in the Paris hospitals, it has been shown that, with very few exceptions, women alone were affected, and that the failures have been much more numerous than the successes. Drs. Demarquay and Giraud Teulon found that the results were very variable in women, and in fifteen instances anæsthesia was produced in but two, and in these the impression was neither lasting nor profound. From the account of the very many attempts made to utilize the procedure, we may affirm that hypnotism is utterly worthless as an anæsthetic agent.

